# REV1 promotes lung tumorigenesis by activating the Rad18/SERTAD2 axis

**DOI:** 10.1038/s41419-022-04567-5

**Published:** 2022-02-03

**Authors:** Yunshang Chen, Xiaohua Jie, Biyuan Xing, Zilong Wu, Xijie Yang, Xinrui Rao, Yingzhuo Xu, Dong Zhou, Xiaorong Dong, Tao Zhang, Kunyu Yang, Zhenyu Li, Gang Wu

**Affiliations:** Cancer Center, Union Hospital, Tongji Medical College, Huazhong University of Science and Technology, Wuhan, 430022 China

**Keywords:** Non-small-cell lung cancer, Oncogenes

## Abstract

REV1 is the central member of the family of TLS polymerases, which participate in various DNA damage repair and tolerance pathways and play a significant role in maintaining genomic stability. However, the role of REV1 in tumors is rarely reported. In this study, we found that the expression of REV1 was significantly upregulated in lung cancer tissues compared with matched adjacent tissues and was associated with poor prognosis. Functional experiments demonstrated that REV1 silencing decreased the growth and proliferation capacity of lung cancer cells. Mechanistically, REV1 upregulated the expression of SERTAD2 in a Rad18-dependent manner, thereby promoting lung carcinogenesis. A novel REV1 inhibitor, JH-RE-06, suppressed lung tumorigenesis in vivo and in vitro and was shown to be safe and well tolerated. Our study confirmed that REV1 is a potential diagnostic marker and therapeutic target for lung cancer and that JH-RE-06 may be a safe and efficient therapeutic agent for NSCLC.

## Introduction

Lung cancer has one of the highest incidences worldwide and is the leading cause of tumor-related death [1, 2]. Non-small cell lung cancer (NSCLC) is the most common histological type of lung cancer, accounting for approximately 85% of cases, while lung adenocarcinoma is the major subtype of NSCLC [3]. Although new diagnosis and treatment methods continue to emerge, many challenges remain in improving the clinical outcomes of lung cancer patients [4]. Therefore, it is urgent to better understand the mechanism of lung tumorigenesis and identify new therapeutic targets.

Translesion DNA synthesis (TLS) is a conservative DNA damage tolerance strategy in both prokaryotes and eukaryotes. TLS relies on a group of unique, low-fidelity TLS polymerases to complete DNA replication in a way that preserves rather than repairs damage [5]. Therefore, TLS increases the mutation rate and the risk of tumorigenesis while maintaining genomic integrity [6, 7]. In addition, as an important part of the DNA damage tolerance pathways, TLS protects tumor cells and allows them to overcome oncogene-induced replication stress (OIRS) during tumor progression [8, 9]. Taken together, these observations indicate that TLS is closely related to carcinogenesis and tumor progression.

REV1 DNA directed polymerase (REV1) is a conserved Y family TLS polymerase that plays a central role in TLS [10, 11]. REV1 preferentially incorporates cytosine across from the damaged site in a template-independent manner via its unique deoxycytidine phosphotransferase activity [12]. Furthermore, REV1 serves as a scaffold to recruit other TLS polymerases and mediates polymerase conversion at stalled replication forks [6, 13]. In addition, REV1 is involved in homologous recombination repair (HR) and nucleotide excision repair (NER) to maintain genome stability [14, 15]. Recently, REV1 has been reported to be related to carcinogenesis and therapeutic resistance. Single-nucleotide polymorphisms (SNPs) in REV1 are involved in the risk of lung squamous cell carcinoma [16]. In mice, REV1 participates in tumorigenesis induced by benzopyrene and N-methyl-N-nitrosourea (MNU) via accumulation of point mutations [17, 18]. Moreover, upregulation of REV1 is associated with cisplatin resistance in ovarian cancer cells and radiotherapy resistance in head and neck squamous cell carcinoma [19, 20]. The above findings suggest that REV1 is a potential oncogene in tumors. Notably, the novel small molecule inhibitor JH-RE-06 recently showed good prospects for clinical application by blocking the function of REV1. Wojtaszek JL et al found that JH-RE-06 can reduce the occurrence of cisplatin-induced mutations and improve the sensitivity of tumor cells to cisplatin. When combined with cisplatin, JH-RE-06 almost completely inhibited tumor growth and significantly increased the survival time of tumor-bearing mice [21]. However, because JH-RE-06 is a new inhibitor, its antitumor function remains to be further explored.

In the present study, we found that REV1 is abnormally upregulated in patients with lung cancer and that overexpression of REV1 is associated with poor prognosis. Targeted genetic or pharmacological inhibition of REV1 was confirmed to hinder lung tumorigenesis by downregulating the Rad18/SERTAD2 signaling axis. Importantly, the REV1 inhibitor JH-RE-06 showed a significant anticancer effect and exhibited good safety and tolerability. These results suggest that REV1 is a potential therapeutic target and that its inhibitor JH-RE-06 is a promising antineoplastic drug.

## Materials and methods

### Cell culture

Human lung carcinoma cell lines (A549, H1299, Calu-1, and SPC-A1) and the normal bronchial epithelial cells HBE were purchased from the American Type Culture Collection (ATCC). All cells were cultured in either DMEM/F12 or RPMI-1640 medium supplemented with 10% fetal calf serum and 100 μg/mL penicillin and streptomycin at 37 °C in 5% CO_2_. All cell lines were regularly tested for contamination with mycoplasma or other pathogens and authenticated using short tandem repeat (STR) profiling.

### Plasmid transfection

The Flag-REV1 and SFB-SERTAD2 plasmids were purchased from Vigene Biosciences. We performed plasmid transfection using Lipofectamine 2000 reagent (Invitrogen). Transfected cells were harvested for subsequent experiments after 24 h.

### Antibodies and reagents

The primary antibodies and reagents used in this study included mouse anti-REV1 (Santa Cruz, sc-393022, 1:100), rabbit anti-Rad18 (Proteintech, 18333-1-AP, 1:500), rabbit anti-RPA32 (Proteintech, 10412-1-AP, 1:500), rabbit anti-GAPDH (Servicebio, GB11002, 1:2000), rabbit anti-SERTAD2 (Abcam, ab272581, 1:500), mouse anti-YY2 (Proteintech, 66839-1-Ig, 1:3000), rabbit anti-FAM84B (Proteintech, 18421-1-AP, 1:1000), rabbit anti-DHRS2 (Proteintech, 15735-1-AP, 1:1000), rabbit anti-TOR2A (Proteintech, 19511-1-AP, 1:500), mouse anti-Flag (Sigma-Aldrich, F1804, 1:1000), rabbit anti-mUb-PCNA (Cell Signaling Technology, #13439, 1:500), rabbit anti-REV7 (Proteintech, 12683-1-AP, 1:500), rabbit anti-REV3 (Abclonal, A10675, 1:500), and JH-RE-06 (Topscience, T15611, China).

### RNA interference

REV1, Rad18 and SERTAD2 were knocked down in A549 and H1299 cells by transfection with the indicated small interfering RNAs (siRNAs). Cells were transfected with siRNAs with Lipofectamine RNAiMAX reagent (Invitrogen) for 48 h. The sequences of the siRNAs were as follows:

Scramble siRNA: 5′-UUCUCCGAACGUGUCACGUTT-3′,

SiREV1#1: 5′-GAACAGUGACGCAGGAAUA-3′,

SiREV1#2: 5′-GCAUCAAAGCUGGACGACU-3′,

SiSERTAD2#1: 5′-GGUCCAUCCAAGGUGUCUUTT-3′,

SiSERTAD2#2: 5′-CCACAUCAUGGAGGUGCUUTT-3′,

SiRad18#1: 5′-CCAGCCAAAUCUCCUGCUUTT-3′, and

SiRad18#2: 5′-GCGUCUUGAAGCUAGUAAATT-3′.

### Western blot analysis

After three washes with cold PBS, cells were homogenized in NETN lysis buffer containing 20 mM Tris-HCl (pH 8.0), 100 mM NaCl, 1 mM EDTA and 0.5% Nonidet P-40 at 4 °C for 30 min and were then centrifuged at 12,000 rpm for 25 min at 4 °C. The supernatant was collected and denatured in 5 × SDS buffer at 100 °C for 10 min. The samples were electrophoresed, and proteins were transferred to PVDF membranes that were activated by wetting in methanol. Membranes were subsequently incubated with the relevant primary antibody overnight at 4 °C and with the secondary antibody the next day. Immunoreactions were detected via enhanced chemiluminescence.

### Real-time qPCR

Total RNA was isolated with an RNA extraction kit (Omega, R6834-01, USA). Reverse transcription was conducted using HiScript III-RT SuperMix for qPCR (Vazyme, R323-01, China). Real-time qPCR was performed using ChamQ SYBR qPCR Master Mix (Vazyme, Q311-02) with GAPDH as the internal reference gene. We utilized the ΔCt method to calculate the relative mRNA levels of target genes. The sequences of the PCR primers are listed in Supplementary Table 1.

### RNA-seq

Cells were harvested for RNA-seq 48 h after being transfected with scramble or REV1-targeting siRNAs. Total RNA was isolated by using TRIzol reagent (Invitrogen) and sent to BGI-Shenzhen, China, for RNA-seq on the BGISEQ-500 platform. The specific process of transcriptome sequencing was as follows: firstly, the purified and fragmented mRNA was reverse transcribed into cDNA, and the product was amplified and enriched by PCR; then Qubit method was used to quantify the PCR product to construct a single-stranded DNA loop (ssDNA loop), which gave the final library. In the process of sequencing, the roller copy technology (RCR) was employed to amplify the fluorescence signal, and the data were read and analyzed through the BGISEQ-500 platform. We have uploaded the original RNA-seq data generated in our study to the Gene Expression Omnibus under registration number GSE183332.

### Cell growth and proliferation assays

Cell proliferation rates were assessed by using cell growth curves and plate colony formation assays. For the growth assays, cells transfected with the indicated siRNAs for 48 h or treated with the drug for 24 h were seeded in six-well plates (1.0 × 10^4^ cells per well) and counted every other day after harvesting with trypsin and resuspension. For the plate colony formation assays, the indicated cells were seeded in six-well plates (500 cells per well) and grown for fourteen days. After being fixed with methanol and stained with crystal violet, colonies containing more than 50 cells were counted.

### EdU incorporation assay

The EdU incorporation assay was performed by using an EdU kit (Beyotime, C0078S, China) according to the instruction manual. In brief, cells were plated in a 96-well plate and harvested the next day. After being incubated with 10 μM EdU for 2 h, cells were fixed with 4% formaldehyde for 15 min and permeabilized with 0.3% Triton X-100 in PBS for 10 min. The samples were then stained with click reaction solution for 30 min and counterstained with DAPI for 10 min. We used a fluorescence microscope to visualize cells and ImageJ software to count cells.

### CCK-8 assay

CCK-8 assay solution (Beyotime, C0037) was utilized to measure the half-maximal inhibitory concentration (IC50) of JH-RE-06. We added CCK-8 assay solution to each well of 96-well plates initially seeded with five thousand cells. After incubation for 1 h, we measured the absorbance at 450 nm by using a spectrophotometer (EnSpire® 2300, USA).

### IHC staining

A lung adenocarcinoma tissue microarray containing carcinoma and paired adjacent noncancerous tissue samples from 77 individual lung adenocarcinoma patients was purchased from Outdo Biotech (Shanghai, China). For IHC staining, paraffin tissue sections were dehydrated and treated with 3% hydrogen peroxide at room temperature for 10 min. After being blocked with 10% goat serum, the sections were incubated with anti-REV1 (Santa Cruz, sc-393022, 1:50), anti-Rad18 (Proteintech, 18333-1-AP, 1:200), anti-RPA32 (Proteintech, 10412-1-AP, 1:400), anti-Ki67 (Abcam, ab16667, 1:1200) and anti-SERTAD2 (Abcam, ab272581, 1:100) antibodies overnight at 4 °C. After being incubated with secondary antibodies, the sections were dehydrated and mounted after counterstaining with hematoxylin for 30 s. All of our IHC results were scored independently by three pathologists using the same scoring criteria by following the principle of blindness. The IHC score was calculated as the product of staining intensity and positive staining area. We defined scores lower than 6 as indicating low expression and scores equal to or greater than 6 as indicating high expression. The staining intensity was graded as follows: 0, negative; 1, weak; 2, moderate; and 3, strong. The positive staining area was scored as follows: 1, 0–25%; 2, 26–50%; 3, 51–75%; and 4, >75%.

### In vivo xenograft mouse model

Female BALB/c nude mice aged four to five weeks were purchased from Changzhou Cavens Laboratory Animal Co., Ltd and were randomly divided into two groups (at least six mice per group according to previous experience). A total of 5 × 10^6^ A549 or H1299 cells were injected into the right axilla. JH-RE-06 dissolved in 10% EtOH, 40% PEG400, and 50% saline was injected directly into the tumors at 1.6 mg/kg when the tumor volume reached 100 mm^3^. Mice were treated every other day. Tumor volumes and mouse weights were measured every three days with blinding. The formula used to calculate the tumor volume was V = (Length × Width^2^). HE staining was used to visualize general tissue morphology.

### Biochemical analysis of peripheral blood

Peripheral blood was obtained from mice via retroorbital bleeding. After being mixed by inversion in an anticoagulant tube, the blood samples were centrifuged at 5000 rpm for 5 min to obtain serum samples. Biochemical indexes were evaluated in the peripheral blood of mice by a biochemical analyzer (Pointcare M4).

### Statistical analysis

All in vitro experiments were repeated three times independently. The data are shown as the means ± SDs or means ± SEMs. The differences between the two groups were analyzed by a t-test (two-tailed), while differences among multiple groups were analyzed by one-way analysis of variance (ANOVA). Overall survival was evaluated using the Kaplan-Meier method. Clinical characteristics of lung cancer patients were assessed by the chi-square test. Statistical correlations of gene expressions were analyzed by Spearman’s analysis. Sample sizes were chosen based on previous experience. All data meet the assumptions of the tests and statistical tests are justified as appropriate. *P* < 0.05 was considered to be statistically significant (*P < 0.05, **P < 0.01, ***P < 0.001).

## Results

### REV1 is overexpressed and indicates poor prognosis in lung cancer

**Fig. 1:**
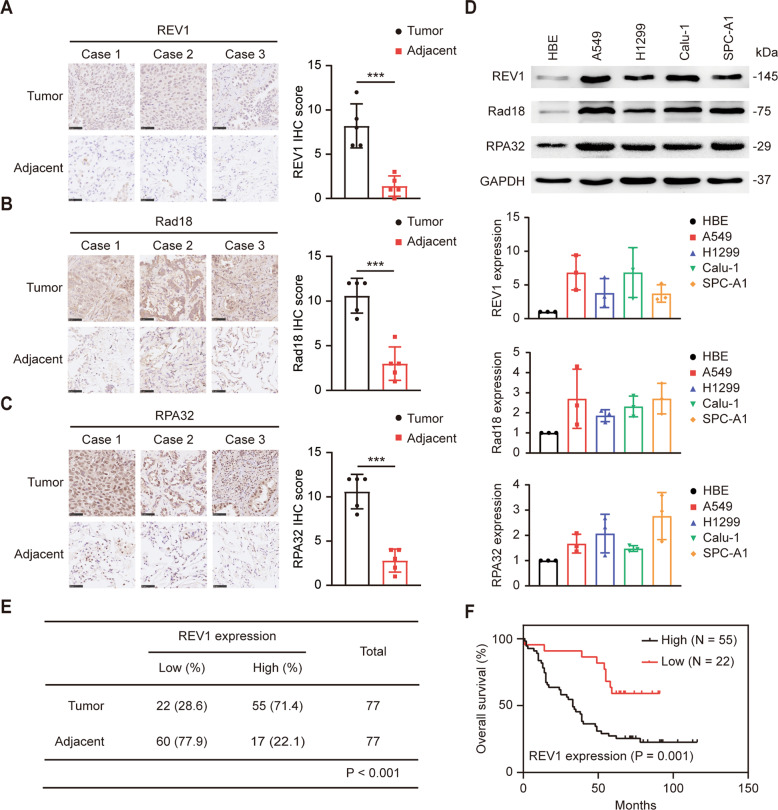
REV1 is overexpressed and indicates poor prognosis in lung cancer. **A**–**C** Representative immunohistochemical staining images and IHC scores of REV1, Rad18, and RPA32 in lung cancer and paracancerous tissues. ***P < 0.001 (*n* = 5). Scale bar: 50 µm. **D** Protein levels of REV1, Rad18 and RPA32 in different cell lines, with the relative expression statistics from three independent experiments. The data are presented as the means ± SDs (*n* = 3). **E** Statistical analysis of REV1 expression in the lung adenocarcinoma tissue microarray. **F** Relationship between the expression of REV1 and the prognosis of patients with lung cancer.

TLS is an error-prone DNA damage tolerance mechanism that can increase both the frequency of mutations and tumorigenesis [22]. The recruitment of RPA32 and Rad18 to stalled replication forks can facilitate the ubiquitination of proliferating cell nuclear antigen (PCNA), which can recruit TLS polymerases such as REV1 to initiate TLS [23, 24]. To explore the role of TLS in lung cancer, we used several publicly available databases and resources including TCGA (https://www.cancer.gov/about-nci/organization/ccg/research/structural-genomics/tcga), UALCAN (http://ualcan.path.uab.edu/) and HPA (https://www.proteinatlas.org/) to search for the expression of key molecules of TLS in lung cancer. Compared with adjacent tissues, REV1, Rad18, RPA32 and REV7 showed higher expression in lung cancer tissues (Supplementary Fig. 1), indicating that TLS may be abnormally activated in lung cancer. Consistent with the results in databases, we found that the expression of REV1, Rad18 and RPA32 was dramatically upregulated in lung adenocarcinoma tissues, as shown by immunohistochemical (IHC) staining of 5 pairs of lung adenocarcinoma and adjacent tissues (Fig. 1A–C). Western blot analysis showed that the expression of REV1, Rad18, RPA32 and REV7 was markedly elevated in several lung cancer cell lines (A549, H1299, Calu-1, and SPC-A1) compared with the normal bronchial epithelial cells HBE (Fig. 1D, Supplementary Fig. 2). Of these proteins, REV1 exhibited the most obvious change in expression. REV1 has been reported to be the central member of the TLS polymerase family and to play an essential role in the assembly and function of polymerases [6, 13]. We further evaluated the expression of REV1 in lung cancer and its relationship with prognosis. Through IHC staining of a lung adenocarcinoma tissue microarray containing 77 pairs of cancerous and paracancerous tissues, we found that the expression of REV1 in lung cancer tissue was significantly higher than that in adjacent tissue (Fig. 1E). Kaplan–Meier (K–M) survival analysis showed that high expression of REV1 predicted a shorter overall survival time in patients with lung cancer (Fig. 1F). Collectively, the above results suggest that TLS is abnormally activated in lung cancer and that REV1, which plays a central role in TLS, is overexpressed and indicates poor prognosis in lung cancer patients.

### REV1 promotes the growth and proliferation of lung cancer cells

**Fig. 2:**
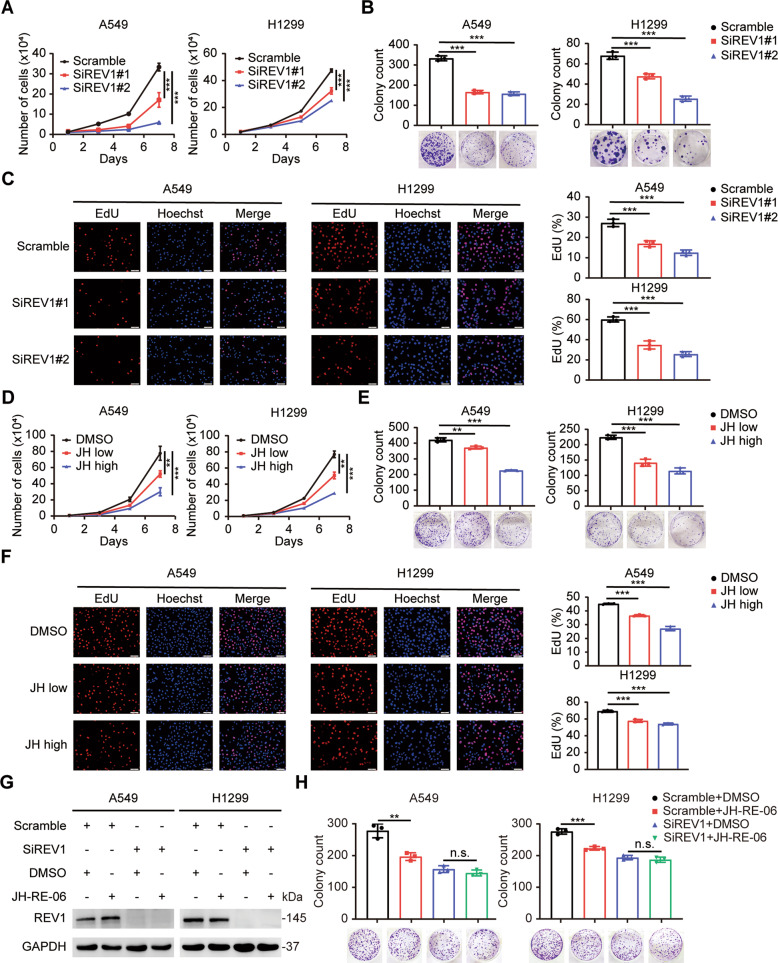
REV1 promotes the growth and proliferation of lung cancer cells. **A** REV1 silencing slowed the growth of A549 and H1299 cells. ***P < 0.001 (*n* = 3). **B** Knockdown of REV1 significantly decreased the colony formation ability of lung cancer cells. ***P < 0.001 (*n* = 3). **C** The proliferation ability of A549 and H1299 cells was evaluated by an EdU incorporation assay. ***P < 0.001 (*n* = 3). Scale bar: 50 μm. **D**–**F** JH-RE-06 inhibited the growth and proliferation ability of A549 and H1299 cells in a concentration-dependent manner, as indicated by cell growth curves, plate colony formation assays and EdU incorporation assays. **P < 0.01, ***P < 0.001 (*n* = 3). Scale bar: 50 μm. **G**, **H** JH-RE-06 inhibits the proliferation ability of lung cancer cells in a REV1-dependent manner. **P < 0.01, ***P < 0.001 (*n* = 3). n.s. indicates no statistically significant difference (*P* > 0.05, *n* = 3).

Given its high expression in lung cancer, we speculated that REV1 may function as an oncogene in the pathogenesis of lung cancer. To test this hypothesis, the expression of REV1 was deregulated in the A549 and H1299 lung cancer cell lines via an RNA interference approach (Supplementary Fig. 3). Silencing endogenous REV1 significantly reduced the growth and colony formation ability of lung cancer cells (Fig. 2A, B). 5-Ethynyl-2’-deoxyuridine (EdU) is a thymidine analog that can be incorporated into synthetic DNA molecules during DNA replication and reflect DNA replication activity [25]. As expected, depletion of REV1 significantly decreased the proportion of EdU-positive cells among lung cancer cells, indicating that the proliferation ability of lung cancer cells was substantially impaired (Fig. 2C). In addition, the small molecule inhibitor JH-RE-06 blocks the function of REV1 by promoting the formation of dimers. In this study, JH-RE-06 was found to be able to suppress the growth and proliferation of lung cancer cells in a concentration-dependent manner (Fig. 2D-F and Supplementary Fig. 4). To determine the specificity of JH-RE-06, we used JH-RE-06 to treat REV1 silencing cells and found that the inhibitory effect of REV1 silencing on the growth and proliferation of lung cancer cells could not be further enhanced, indicating that JH-RE-06 functions in a REV1-dependent manner (Fig. 2G, H). In addition, to exclude the effect of JH-RE-06 on the expression of REV7 and REV3, the protein level of REV7 and REV3 was detected after JH-RE-06 treatment. The results showed that JH-RE-06 did not affect the expression of REV7 and REV3 (Supplementary Fig. 5). Overall, we can conclude that JH-RE-06 is a selective inhibitor of REV1. Both REV1 silencing and JH-RE-06 treatment can inhibit the growth and proliferation of lung cancer cells in vitro.

### The REV1 inhibitor JH-RE-06 suppresses lung tumorigenesis in vivo

**Fig. 3:**
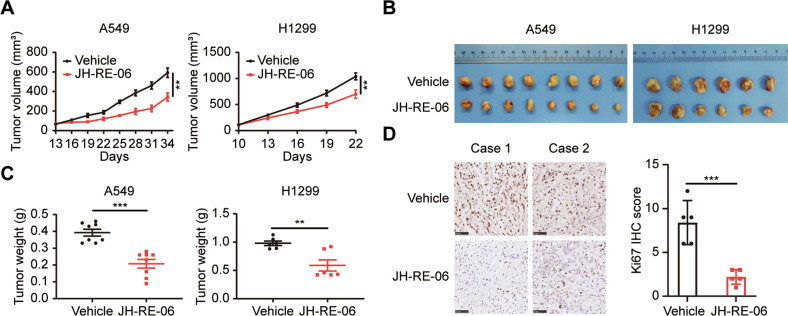
The REV1 inhibitor JH-RE-06 suppresses lung tumorigenesis in vivo. **A** Xenograft tumor growth curves in the two groups. Tumor volumes were measured every three days. The data are shown as the mean tumor volumes ± SEMs. **P < 0.01 (*n* = 8 or 6 mice per group). **B** Pictures of xenografts formed from A549 and H1299 cells with the indicated treatment. **C** Weights of xenograft tumors. **P < 0.01, ***P < 0.001 (*n* = 8 or 6 mice per group). **D** Representative images of IHC staining for Ki67 in xenograft tumors in the two groups and statistical histograms of the IHC score. ***P < 0.001 (*n* = 5). Scale bar: 50 µm.

To explore whether JH-RE-06 can also inhibit lung tumorigenesis in vivo, we established xenograft mouse models by implanting human lung cancer A549 and H1299 cells into T cell-deficient athymic nude mice. Mice in the treated and control groups were treated with JH-RE-06 and vehicle, respectively, by intratumoral injection. The growth of xenograft tumors was impaired, the tumor sizes and weights were decreased, and the survival time was significantly increased in the JH-RE-06 group compared with the control group (Fig. 3A–D). A primary limitation in the clinical use of drugs is their safety and off-target effects; thus, we conducted a series of in-depth safety evaluations in the drug administration model. We found there was no significant difference in body weight between the two groups (Supplementary Fig. 6A). In addition, Hematoxylin and eosin (HE) staining showed that JH-RE-06 did not cause significant pathological damage to important organs in mice, including the heart, liver, spleen, lung and kidney (Supplementary Fig. 6B). Moreover, we comprehensively assessed biochemical indexes (such as albumin (ALB), alanine aminotransferase (ALT), blood urea nitrogen (BUN), serum creatinine (Scr), amylase (AMY), glucose (Glu), et al.) in peripheral blood and found no obvious abnormalities between the two groups (Supplementary Fig. 6C). Considering the above results, we concluded that JH-RE-06 can also suppress lung tumorigenesis in vivo with good safety and no toxic side effects, predicting its promising prospects for clinical application.

### REV1 regulates the expression of SERTAD2 in lung cancer cells

**Fig. 4:**
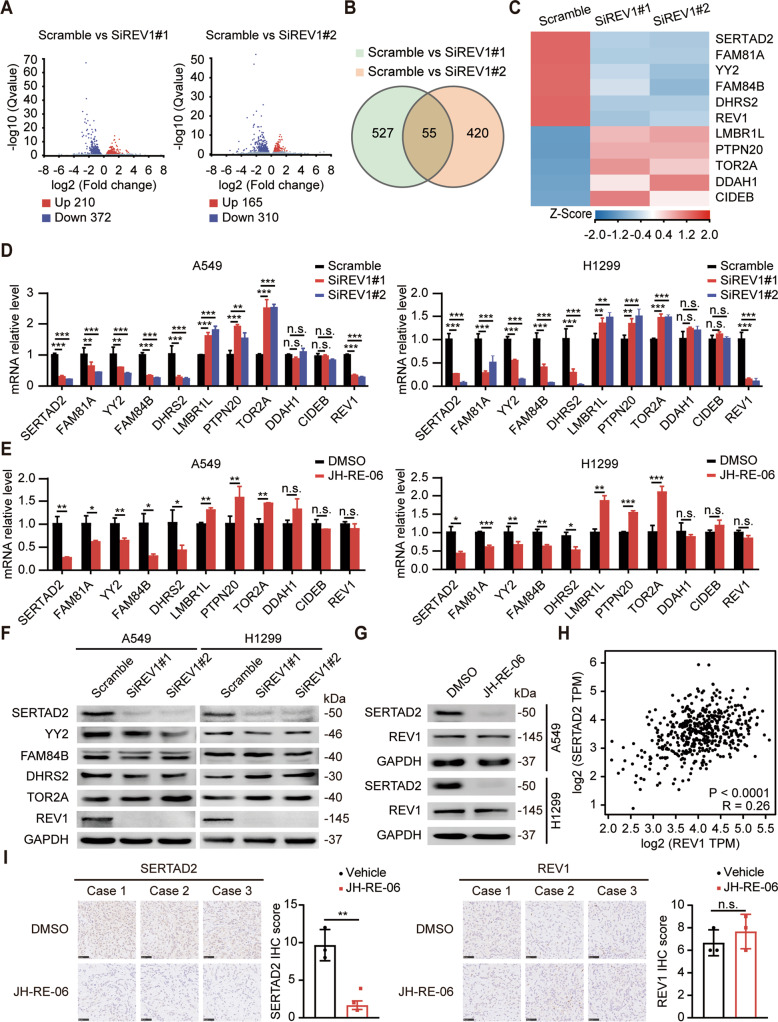
REV1 regulates the expression of SERTAD2 in lung cancer cells. **A** Volcano plots showing differentially expressed genes in REV1-depleted vs. scramble siRNA-transfected A549 cells. **B** Venn diagram showing the number of genes in each indicated set. **C** Clustering heatmap of the ten most deregulated genes affected by REV1 silencing. **D** The mRNA levels of the indicated molecules in scramble siRNA-transfected and REV1-silenced cells were measured by PCR. **P < 0.01, ***P < 0.001, n.s. *P* > 0.05 (*n* = 4). **E** The mRNA level of the indicated molecules in DMSO-treated and JH-RE-06-treated cells was examined by PCR. *P < 0.05, **P < 0.01, ***P < 0.001, n.s. *P* > 0.05 *(n* = 4). **F** The protein level of the indicated molecules in scramble siRNA-transfected and REV1-silenced cells was measured by Western Blot (*n* = 3). **G** The protein level of the indicated molecules in DMSO-treated and JH-RE-06-treated cells was examined by Western Blot (*n* = 3). **H** Scatter plot showing the correlation between REV1 and SERTAD2 expression in lung adenocarcinoma samples available in the GEPIA database. **I** Representative images of IHC staining for SERTAD2 in DMSO-treated and JH-RE-06-treated xenograft tumors and statistical histograms of the IHC score. **P < 0.01, n.s. *P* > 0.05 (*n* = 3). Scale bar: 50 µm.

To clarify the specific mechanisms of REV1-induced lung tumorigenesis, we conducted RNA sequencing (RNA-seq) after knockdown of REV1 with two different targeted small interfering RNA (siRNA) sequences. The two siRNAs caused changes in the expression levels of 582 and 475 genes compared with those in the control group respectively (Fig. 4A), of which 55 genes overlapped (Fig. 4B). We selected ten most deregulated genes among those whose expression levels changed more than twice in both siRNAs, including SERTAD2, FAM81A, YY2, FAM84B, DHRS2, LMBR1L, PTPN20, TOR2A, DDAH1 and CIDEB (Fig. 4C). The mRNA level of these molecules was detected by real-time quantitative polymerase chain reaction (qPCR) in scrambled and REV1-silenced lung cancer cells. All genes except DDAH1 and CIDEB showed a consistent change with the results of sequencing (Fig. 4D). In addition, we used JH-RE-06 to inhibit the function of REV1 to verify the changes in the above genes at the mRNA level (Fig. 4E). It is reported that SERTAD2, YY2, FAM84B, DHRS2 and TOR2A are closely related to tumorigenesis, so we detected the protein level of these five molecules in REV1-deleted cells. The results showed that the expression of SERTAD2 was dramatically downregulated with the silencing of REV1, while the protein level of other molecules showed no significant change (Fig. 4F). Moreover, JH-RE-06 can also lead to a remarkable downregulation of SERTAD2 (Fig. 4G), indicating that both genetic and pharmacological targeting of REV1 can affect the expression of SERTAD2. Then, we predicted the correlation between the expression of REV1 and SERTAD2 in lung adenocarcinoma with the GEPIA database (http://gepia.cancer-pku.cn/) and found a positive association (Fig. 4H). As shown in Fig. 4I, the immunohistochemical staining intensity of SERTAD2 in subcutaneous tumors in the JH-RE-06 group was significantly lower than that in the control group. These results indicated that SERTAD2 was a possible downstream target of REV1 and was modulated by REV1 at both the transcriptional and translational levels. SERTAD2 is a transcriptional coregulator that is overexpressed in various human tumors and promotes tumorigenesis in nude mice by upregulating E2F response genes [26]. We explored the expression of SERTAD2 by using TCGA, UALCAN and HPA database and found that SERTAD2 was highly expressed in lung cancer compared with paracancerous tissues, indicating that SERTAD2 may be an oncoprotein in lung cancer (Supplementary Fig. 7A–C). In our follow-up experiments, SERTAD2 silencing was confirmed to inhibit the proliferation of lung cancer cells through colony formation and EdU incorporation assays (Supplementary Fig. 7D–F). Considering these results collectively, we proposed that SERTAD2 is an oncogene in lung cancer and REV1 may promote lung tumorigenesis by regulating the expression of SERTAD2.

### The inhibitory effect of REV1 silencing on the proliferation of lung cancer cells is partially dependent on SERTAD2

**Fig. 5:**
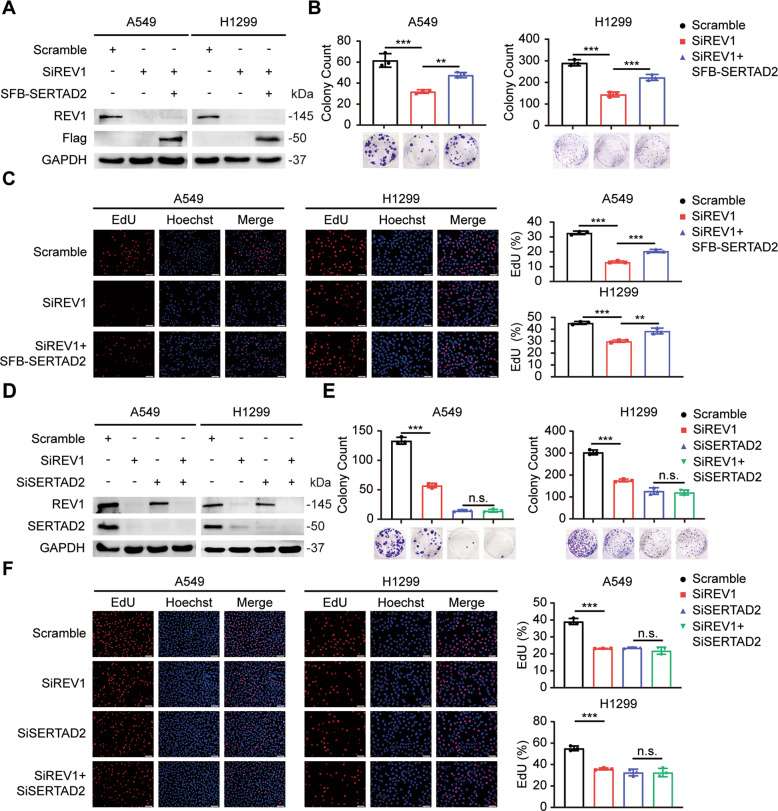
The inhibitory effect of REV1 silencing on the proliferation of lung cancer cells is partially dependent on SERTAD2. **A** A549 and H1299 cells transfected with the indicated siRNAs and SFB-SERTAD2 were harvested and analyzed by Western blot (*n* = 3). **B** Cells transfected with the indicated siRNAs and SFB-SERTAD2 were seeded in a six-well plate in triplicate and grown for two weeks. Representative pictures of colony formation in each group and quantitative analysis of the colony counts are shown. **P < 0.01, ***P < 0.001 (*n* = 3). **C** EdU incorporation assays were used to test the proliferation of cells transfected with the indicated siRNAs and SFB-SERTAD2. **P < 0.01, ***P < 0.001 (*n* = 3). Scale bar: 50 μm. **D** A549 and H1299 cells transfected with the indicated siRNAs were harvested and analyzed by Western blot (*n* = 3). **E** Colony formation assays were conducted to examine the proliferation ability of cells transfected with the indicated siRNAs. ***P < 0.001, n.s. *P* > 0.05 (*n* = 3). **F** Representative fluorescence images of each group transfected with the indicated siRNAs from EdU incorporation assays and statistical analysis results are presented. ***P < 0.001, n.s. *P* > 0.05 (*n* = 3). Scale bar: 50 μm.

To determine the role of SERTAD2 in REV1-mediated tumor promotion, we carried out a series of rescue experiments. First, exogenous SERTAD2 was overexpressed in REV1-depleted A549 and H1299 cells (Fig. 5A). As shown in Fig. 5B, C, SERTAD2 partially reversed the decrease in proliferation induced by REV1 silencing in lung cancer cells. In addition, we transfected siRNAs targeting REV1, SERTAD2 or both into the two cell lines (Fig. 5D). We found that knockdown of REV1 or SERTAD2 markedly suppressed the proliferation of lung cancer cells. However, REV1 knockdown had little impact on the proliferation of SERTAD2-silenced cells (Fig. 5E, F). Overall, the above results suggest that the effect of REV1 on the proliferation of lung cancer cells may be achieved mainly via modulation of SERTAD2.

### The regulatory effect of REV1 on SERTAD2 is partially dependent on Rad18

**Fig. 6:**
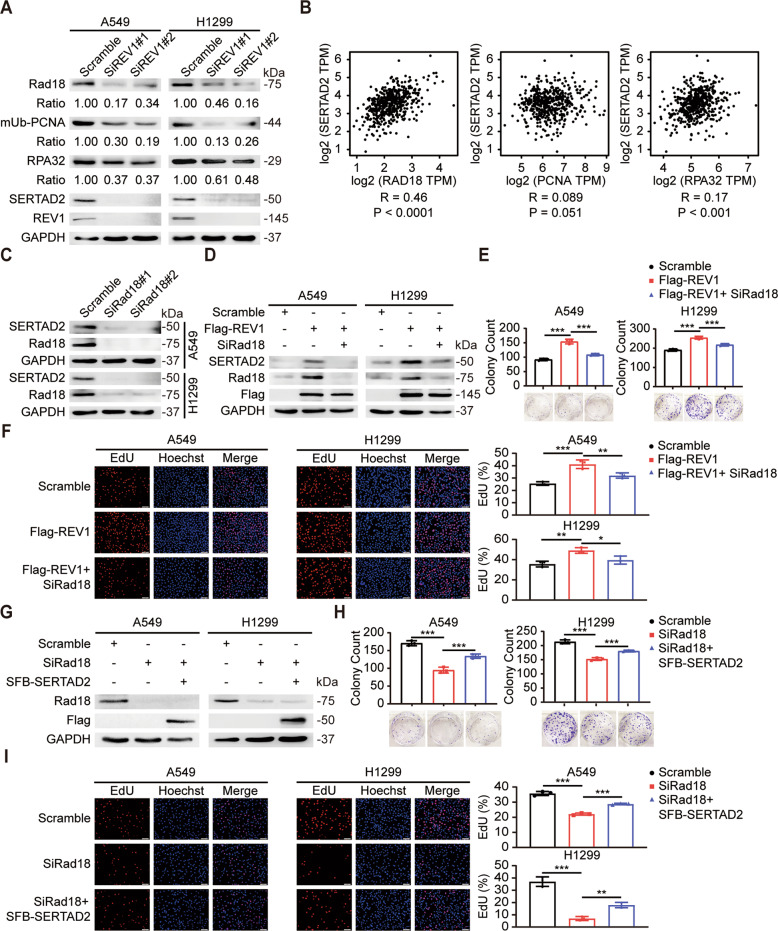
The regulatory effect of REV1 on SERTAD2 is partially dependent on Rad18. **A** Proteins from REV1-silenced A549 and H1299 cells were harvested and examined by Western blot (*n* = 3). **B** Scatter plots of SERTAD2 expression vs. Rad18, PCNA, and RPA32 expression in lung adenocarcinoma samples acquired from the GEPIA database. **C** The protein level of SERTAD2 was decreased in Rad18-depleted cells (*n* = 3). **D** A549 and H1299 cells transfected with SiRad18 and Flag-REV1 were harvested and examined by Western blot (*n* = 3). **E** Cells transfected with SiRad18 and Flag-REV1 were harvested for colony formation assays. Representative pictures of the indicated groups and quantitative analysis of the colony counts are shown. ***P < 0.001 (*n* = 3). **F** EdU incorporation assays were used to test the proliferation of cells transfected with SiRad18 and Flag-REV1. Representative pictures of the indicated groups and statistical analysis results are presented. *P < 0.05, **P < 0.01, ***P < 0.001 (*n* = 3). Scale bar: 50 μm. **G**–**I** A549 and H1299 cells transfected with SiRad18 and SFB-SERTAD2 were harvested for cell proliferation assays. Representative pictures of the indicated groups and the statistical analysis results are shown. **P < 0.01, ***P < 0.001 (*n* = 3). Scale bar: 50 μm.

Since TLS is abnormally activated in lung cancer and REV1 is the central member of the TLS polymerase family, we speculated that the regulatory effect of REV1 on SERTAD2 is related to the biological process of TLS. As mentioned before, the expression of SERTAD2 was significantly downregulated in REV1-silenced A549 and H1299 lung cancer cells. Interestingly, we found that important molecules in the initiation of TLS, including Rad18, monoubiquitinated (mUb)-PCNA and RPA32, were also downregulated to varying degrees (Fig. 6A). The GEPIA database prediction results showed positive correlations between the expression of Rad18, PCNA, RPA32 and that of SERTAD2 in lung adenocarcinoma; indeed, the correlation between Rad18 and SERTAD2 was the most significant (*R* = 0.46, *P* < 0.0001) (Fig. 6B). Therefore, we surmised that the regulation of SERTAD2 by REV1 may be related to Rad18. To verify this hypothesis, we knocked down the expression of Rad18 and found that the expression of SERTAD2 was obviously downregulated (Fig. 6C). The results of subsequent rescue experiments showed that Rad18 silencing partially reversed the upregulation of SERTAD2 expression induced by REV1 overexpression (Fig. 6D). Functional experiments suggested that the enhancement of lung cancer cell proliferation induced by REV1 overexpression was reversed by Rad18 deletion (Fig. 6E, F). In addition, overexpression of SERTAD2 partially restored the inhibitory effect of Rad18 silencing on the proliferation of lung cancer cells (Fig. 6G–I). Accordingly, our results demonstrated that the regulatory effect of REV1 on SERTAD2 is partially dependent on Rad18. REV1 promotes lung tumorigenesis by activating Rad18/SERTAD2 signaling axis in lung cancer cells.

## Discussion

In the present study, we first proposed the important role of REV1 in lung carcinogenesis and revealed the potential molecular mechanism. We found that REV1 is highly expressed in lung cancer and that a high expression level of REV1 predicts poor prognosis. In addition, we demonstrated that REV1 upregulates the expression of the transcriptional coregulator SERTAD2 in a Rad18-dependent manner, which in turn facilitates lung tumorigenesis. A novel small molecule inhibitor of REV1, JH-RE-06, was shown to inhibit the growth and proliferation of lung cancer cells in vivo and in vitro with a manageable safety profile.

REV1, the central member of the TLS polymerase family, mediates the error-prone DNA damage tolerance mechanism and participates in various endogenous and exogenous DNA damage responses [27, 28]. REV1 maintains genomic stability at the cost of increasing point mutations due to its characteristics of low fidelity and low catalytic efficiency [29, 30]. In our study, REV1 was revealed to be a novel oncoprotein in lung cancer. Through IHC staining of lung adenocarcinoma and adjacent tissue samples and Western blot analysis of multiple lung cancer cell lines, we found that many important molecules in TLS (REV1, Rad18 and RPA32) were abnormally overexpressed in lung cancer. Considering the indispensable role of REV1 in TLS, we further explored the clinical relevance of REV1. Our results indicated that REV1 is overexpressed in lung cancer and that high expression of REV1 is associated with poor clinical outcomes in lung cancer patients. Functionally, we demonstrated that inhibition of REV1 can significantly reduce the growth and proliferation of lung cancer cells both in vivo and in vitro. From these results, we can conclude that TLS is abnormally activated in lung cancer. Specifically, the central polymerase in TLS, REV1, is abnormally overexpressed and exerts oncogenic effects in lung cancer. Considering its crucial role as an oncogenic driver, REV1 may constitute a promising prognostic marker and therapeutic target for NSCLC.

To further explore the molecular mechanisms underlying the biological role of REV1 in lung cancer, RNA-seq and verification experiments were performed to identify downstream targets. We found that the transcriptional coregulator SERTAD2 may be a primary effector molecule of REV1. Subsequent rescue experiments confirmed our hypothesis that the regulatory effect of REV1 on the proliferation of lung cancer cells is partially dependent on SERTAD2. SERTAD2 (also called TRIP-Br2) is a member of the TRIP-Br/SERTAD family of transcriptional coregulators in mammals. As a proto-oncogene, it regulates the E2F/DP transcriptional pathway and promotes tumorigenesis by upregulating key genes in the E2F response, including Cyclin E, Cyclin A2, CDC6 and DHFR [26]. However, the mechanism by which REV1 modulates the expression of SERTAD2 remains unclear. Recruitment of REV1 to the stalled replication fork, which is dependent on mUb-PCNA, is an important part of TLS activation [31]. The E3 ubiquitin ligase Rad18 induces the monoubiquitination of PCNA and is recruited to chromatin through its interaction with RPA32 [32–34]. Therefore, recruitment of REV1 to DNA damage sites is regulated by Rad18, mUb-PCNA and RPA32. Interestingly, we found that REV1 can modulate the expression of Rad18, mUb-PCNA and RPA32. In REV1-silenced lung cancer cells, the expression level of SERTAD2 was significantly reduced, and the expression levels of Rad18, mUb-PCNA and RPA32 were also reduced to varying degrees. In addition, bioinformatics analysis with the GEPIA database indicated that the expression of SERTAD2 was strongly positively correlated with that of Rad18 (R = 0.46, *P* < 0.0001). Therefore, we speculated that the regulatory effect of REV1 on SERTAD2 may depend on Rad18. We thus conducted a series of rescue experiments and found that knocking down Rad18 with siRNAs partially reversed the upregulation of SERTAD2 expression induced by REV1 overexpression. Functionally, the acceleration of lung cancer cell proliferation induced by overexpression of REV1 was partially reversed by deletion of Rad18. Furthermore, overexpression of SERTAD2 partially reversed the suppression of lung cancer cell proliferation induced by silencing of Rad18. The above experiments showed that REV1 upregulates the expression of SERTAD2 in a Rad18-dependent manner, providing new insights into the mechanisms by which REV1 promotes lung tumorigenesis.

A newly synthesized small molecule inhibitor, JH-RE-06, can act as a “glue” to “stick” two REV1 proteins together to form a nonfunctional dimer, thereby inhibiting the function of REV1 [21]. JH-RE-06 has been reported to increase sensitivity to cisplatin therapy by enhancing the senescence phenotype of tumor cells [35]. In our study, the results of in vitro cell experiments indicated that JH-RE-06 impaired the growth and proliferation of many kinds of lung cancer cells. Similarly, the effect of JH-RE-06 was verified in vivo. The growth of xenograft tumors was slowed, the tumor size and weight were reduced, and the survival time was significantly increased in the JH-RE-06 group compared with the control group. More importantly, we also comprehensively assessed the potential toxicity of JH-RE-06 and found no significant difference in the body weight between the two groups. In addition, we did not find obvious pathological damage in the important organs (heart, liver, spleen, lung, and kidney) in the JH-RE-06 group compared with the control group by HE staining. Moreover, biochemical testing was carried out on the peripheral blood of the two groups of mice, and the results were visualized by cluster analysis. We found no obvious abnormalities in biochemical indexes of important organs, such as albumin (ALB), alanine aminotransferase (ALT), blood urea nitrogen (BUN), serum creatinine (Scr), amylase (AMY), glucose (Glu), et al. Consequently, our study confirmed that JH-RE-06 holds promise for clinical translation since it not only exerts antitumor effects in lung cancer but also has good safety and no toxicity in vivo.

In summary, our results identified the oncogenic role of REV1 in lung cancer, suggesting that REV1 may constitute a new prognostic marker and therapeutic target for NSCLC. Mechanistically, we confirmed that REV1 promotes pulmonary carcinogenesis by activating the Rad18/SERTAD2 axis. More importantly, the REV1 inhibitor JH-RE-06 may be an attractive potential antitumor agent due to its inhibitory effects on the proliferation of lung cancer cells and low toxicity in vivo.

## Supplementary information

Supplementary Fig. 1

Supplementary Fig. 2

Supplementary Fig. 3

Supplementary Fig. 4

Supplementary Fig. 5

Supplementary Fig. 6

Supplementary Fig. 7

Supplemental Files

Reproducibility Checklist

Author Contribution Statement

## Data Availability

All data generated or analyzed during this study are included in this article and its supplementary information files.
