# Supplementary figures and images for: REV1 promotes lung tumorigenesis by activating the Rad18/SERTAD2 axis
Source: Cell Death Dis. 2022 Feb 3;13(2):110. doi: 10.1038/s41419-022-04567-5 (PMC8814179; doi:10.1038/s41419-022-04567-5)

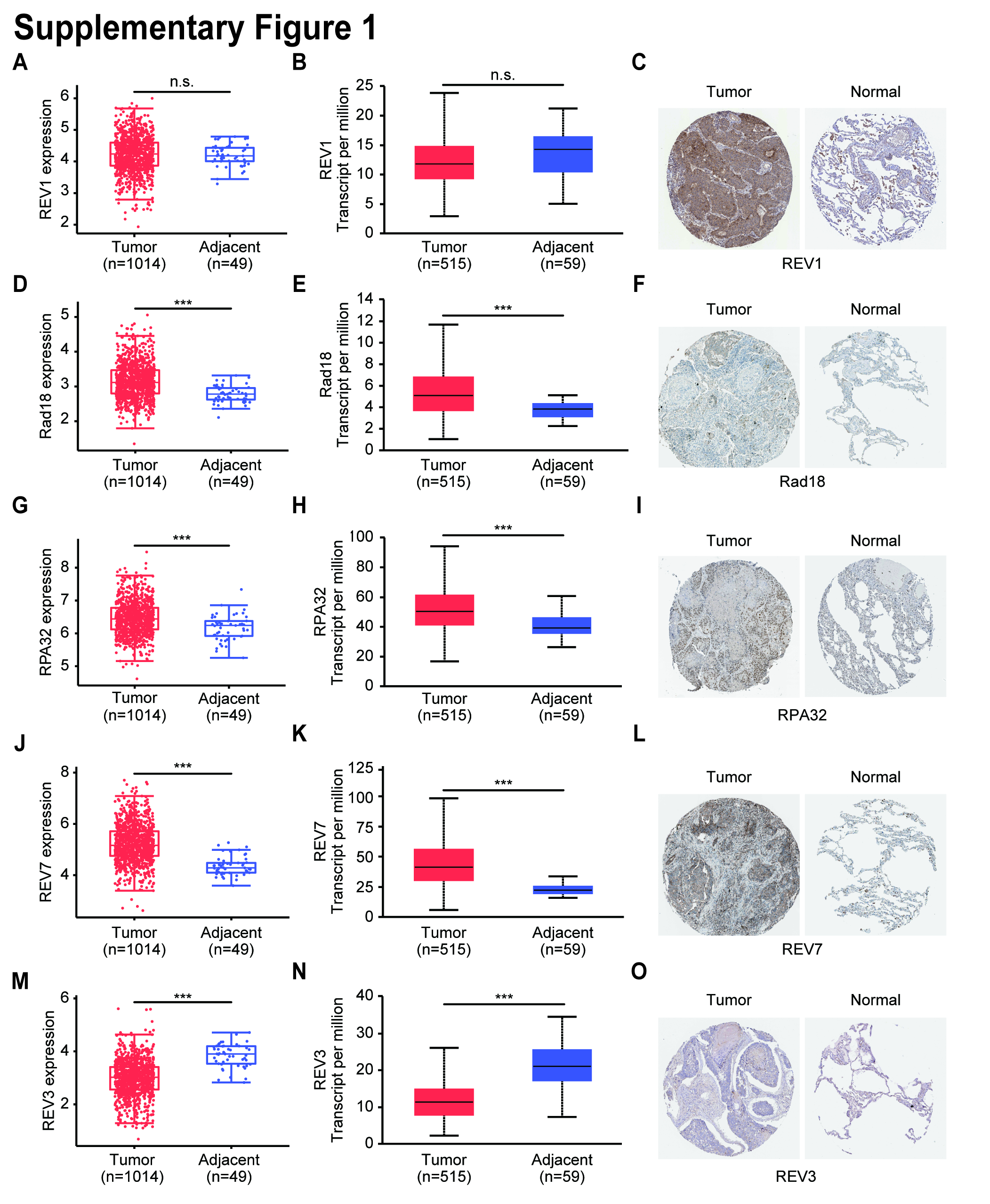

Supplement: Supplementary file 1 — Supplementary Fig. 1 [file 41419_2022_4567_MOESM1_ESM.tif]

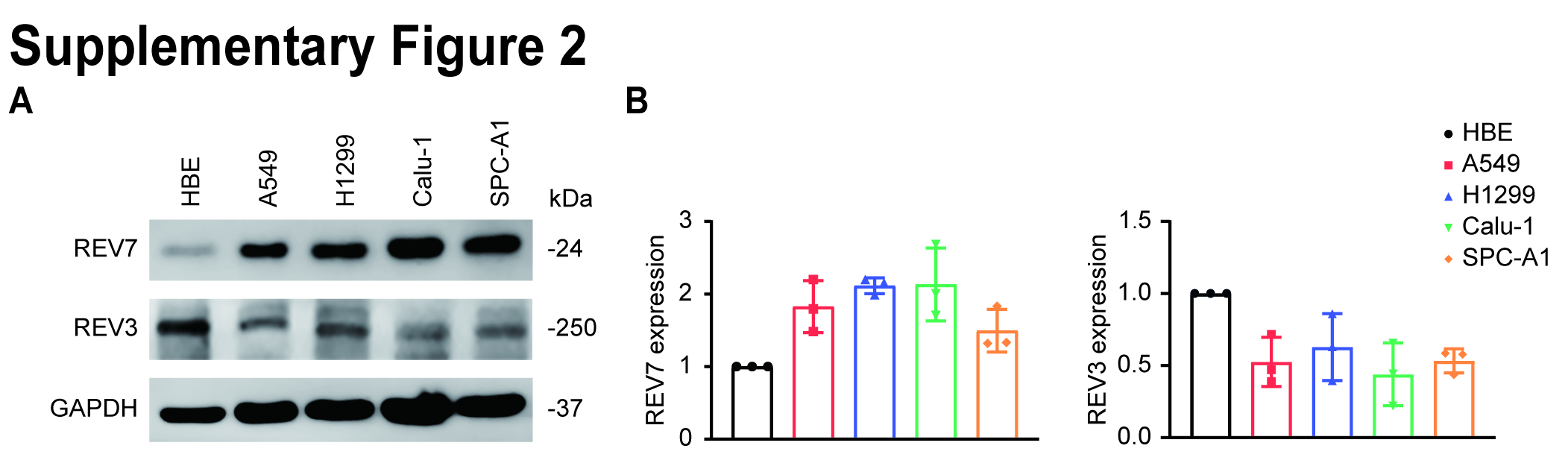

Supplement: Supplementary file 2 — Supplementary Fig. 2 [file 41419_2022_4567_MOESM2_ESM.tif]

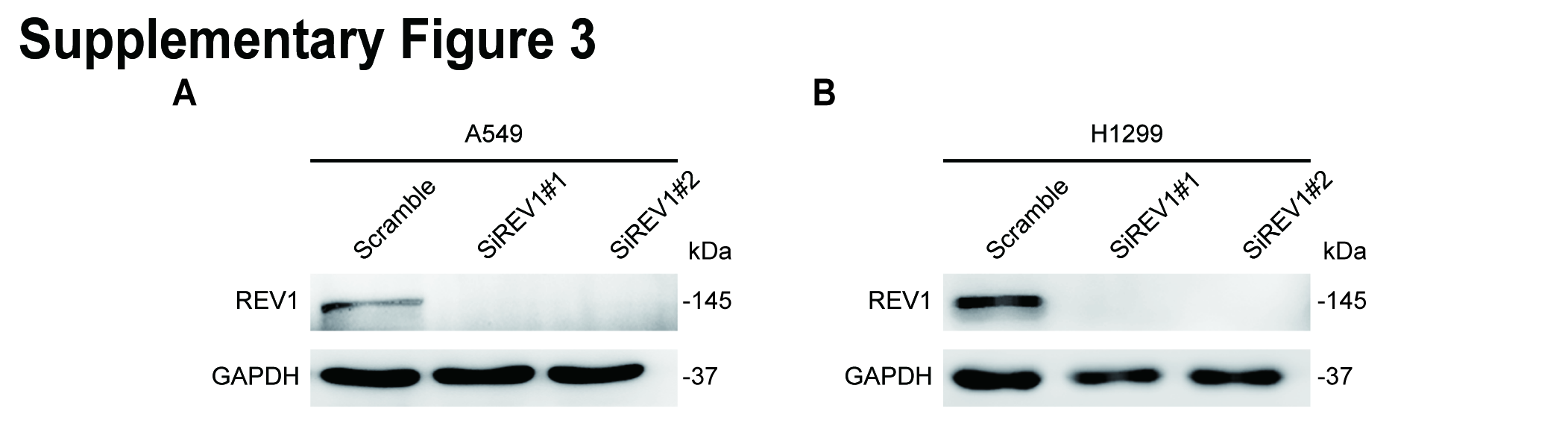

Supplement: Supplementary file 3 — Supplementary Fig. 3 [file 41419_2022_4567_MOESM3_ESM.tif]

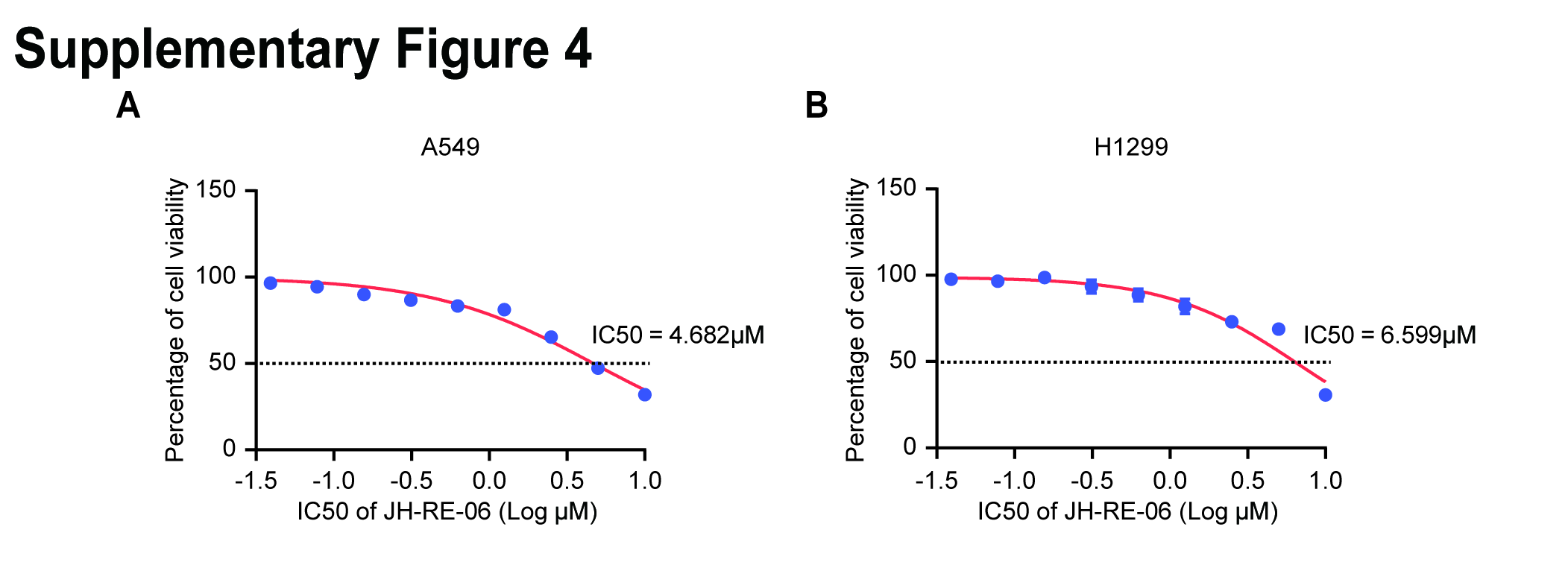

Supplement: Supplementary file 4 — Supplementary Fig. 4 [file 41419_2022_4567_MOESM4_ESM.tif]

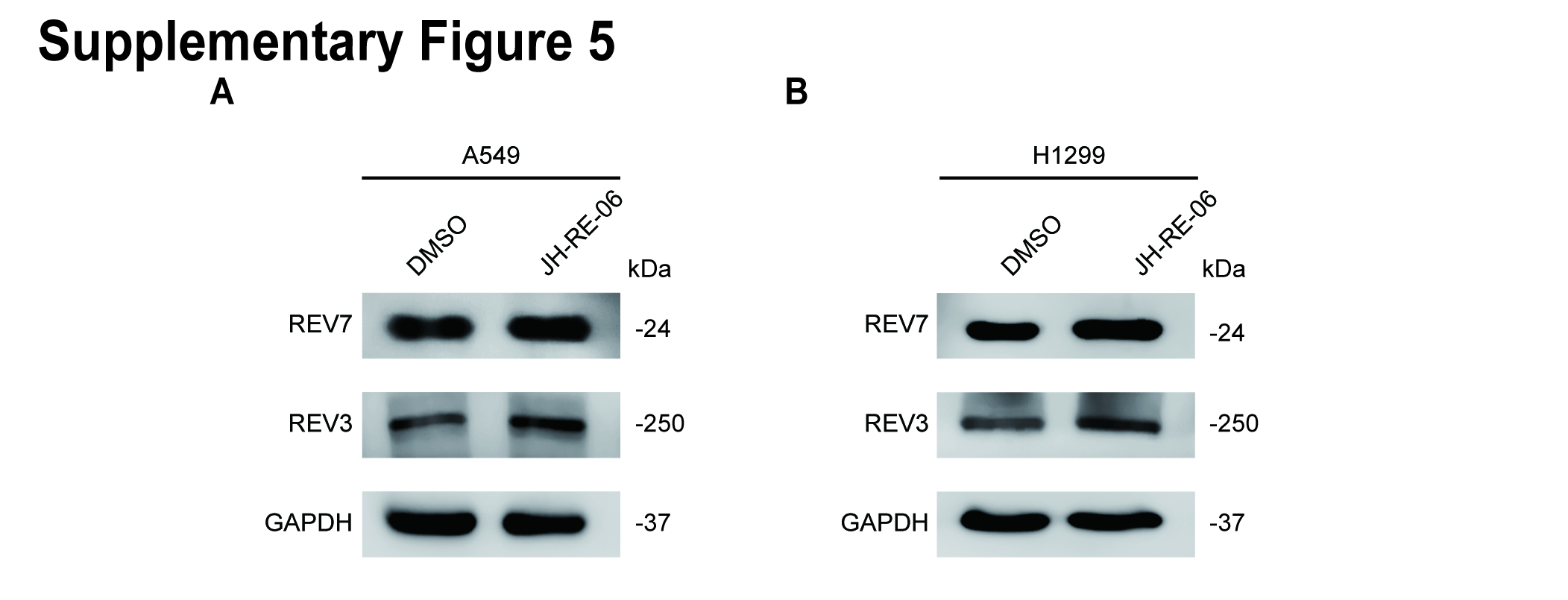

Supplement: Supplementary file 5 — Supplementary Fig. 5 [file 41419_2022_4567_MOESM5_ESM.tif]

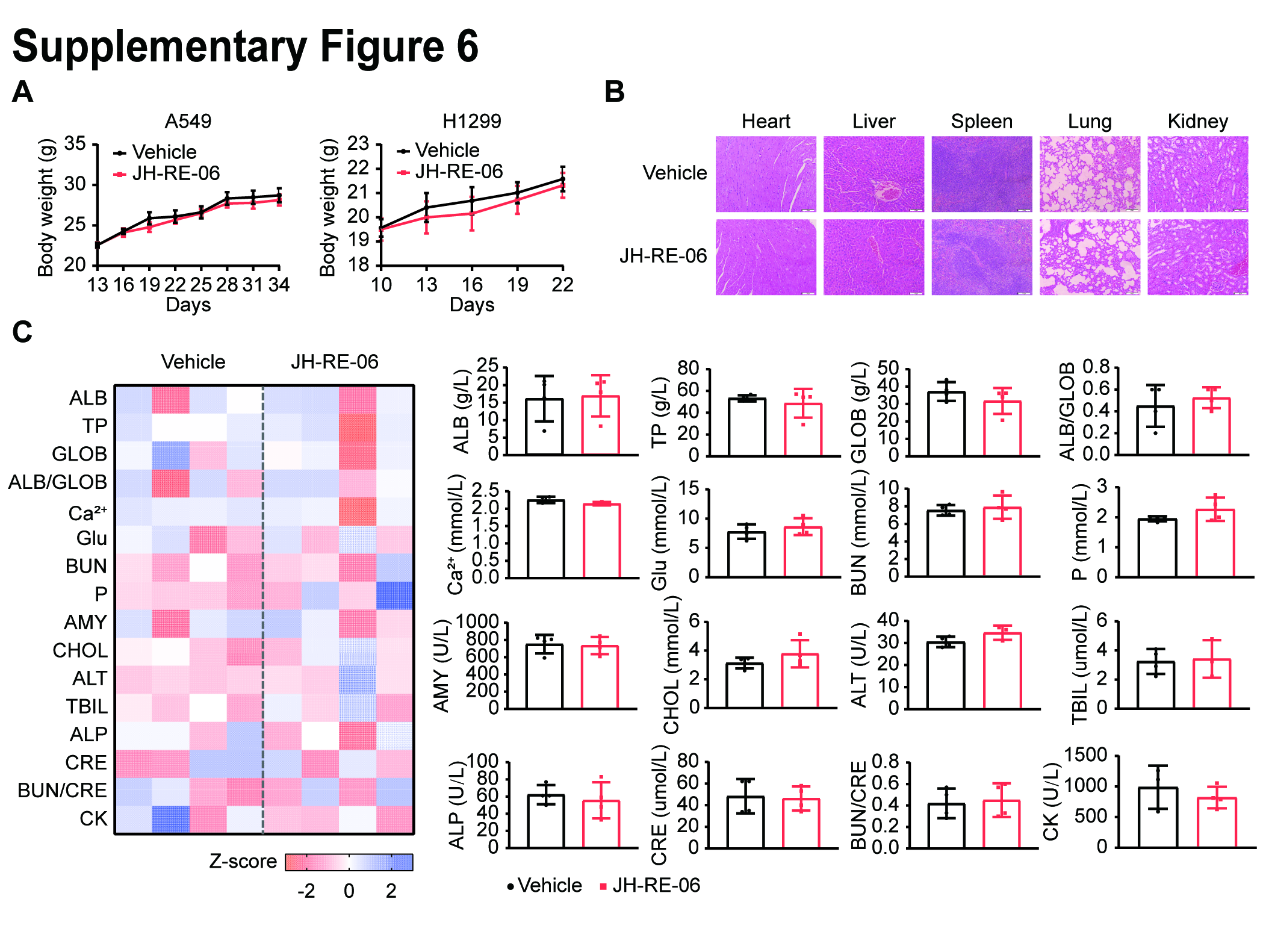

Supplement: Supplementary file 6 — Supplementary Fig. 6 [file 41419_2022_4567_MOESM6_ESM.tif]

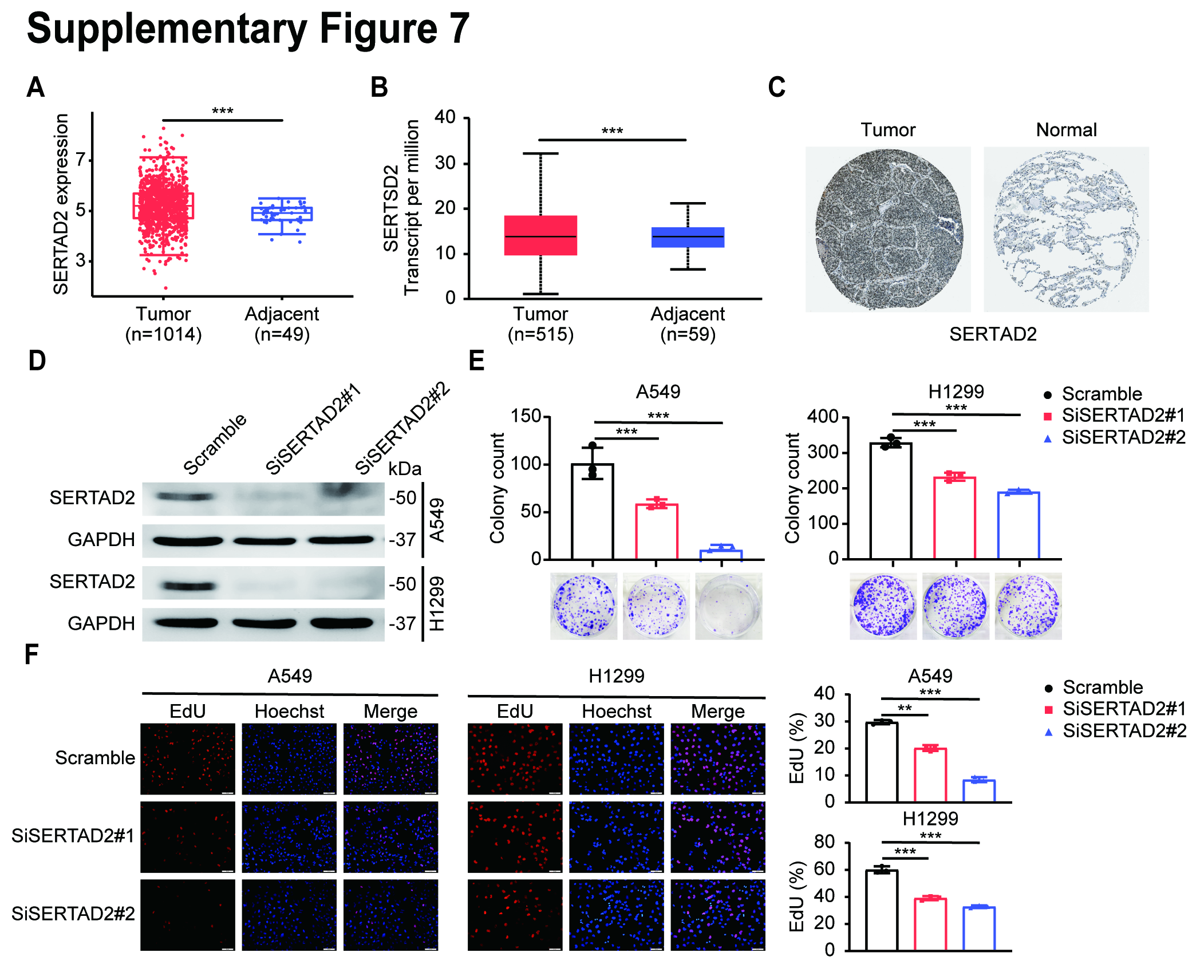

Supplement: Supplementary file 7 — Supplementary Fig. 7 [file 41419_2022_4567_MOESM7_ESM.tif]
