# Supplementary material for: REV1 promotes lung tumorigenesis by activating the Rad18/SERTAD2 axis
Source: Cell Death Dis. 2022 Feb 3;13(2):110. doi: 10.1038/s41419-022-04567-5 (PMC8814179; doi:10.1038/s41419-022-04567-5)
Supplement: Supplementary file 8 — Supplemental Files [file 41419_2022_4567_MOESM8_ESM.docx]

| **Genes** | **Sequences (5'--3')** |
| --- | --- |
| REV1 | F: CAGTCGCCCATCAGTTCAGT |
|  | R: CTTTGTGCTCCTCTTCGGTG |
| SERTAD2 | F: GACAGACTTGACCCTGGATGA |
|  | R: TGTCCCTGATGAGGAAGTGC |
| FAM81A | F: GAAGAGTGGCAAGATGTG |
|  | R: TTTCGTTTCCAAGATAAGT |
| YY2 | F: GACCAGGAAATGCTTATGTT |
|  | R: GTGCTCGTCTTCAATGCTAT |
| FAM84B | F: GAGGTGGAATGCTCCGTGTT |
|  | R: GGTTCTCGGGCGTGTAGGT |
| DHRS2 | F: AGCCAGTTGCTGCCCTACAT |
|  | R: CTGGAACCACGCAGTTTACC |
| LMBR1L | F: GGATTTCCCTCTTTATTGC |
|  | R: GGGTAAGTTCTGCCGATA |
| PTPN20 | F: GAATCTGCCTGGTGAGTT |
|  | R: TTGATGTAGTCCTTGCTTT |
| TOR2A | F: CACCACTTTTCTCCCGTCC |
|  | R: GGGGCATCTTGTCCATCTCAT |
| DDAH1 | F: ATGGCGGAGATGTTTTAT |
|  | R: ACTGGCACTGTGGAGACT |
| CIDEB | F: CCAAGCACAGCAAGGACATC |
|  | R: GACATTCAGGCTGCCAAAGAG |
| GAPDH | F: AGAAGGCTGGGGCTCATTTG |
|  | R: AGGGGCCATCCACAGTCTTC |

F, forward primer; R, reverse primer.

**Supplementary Table 1** Sequences of primers used for Real-time quantitative PCR.

**Supplementary figure legends**

**Supplementary Fig. 1 The expression level of TLS molecules in public tumor databases (TCGA, UALCAN, and HPA).** REV1 (**A-C)**. Rad18 (**D-F**). RPA32 (**G-I**). REV7 (**J-L**). REV3 (**M-O**). **** P* < 0.001, n.s. *P* > 0.05.

**Supplementary Fig. 2 The protein level of REV7 and REV3 in lung cancer cells.** **A** The expression of REV7 and REV3 in different cell lines was detected by Western Blot. **B** The REV7 and REV3 relative expression statistics from three independent experiments. The data are presented as the means ± SDs (n = 3).

**Supplementary Fig. 3 REV1 can be effectively silenced via two siRNAs.** A549 and H1299 cells were transfected with siRNAs targeting REV1 for 48 h, and cellular proteins were collected for Western blot analysis (n = 3).

**Supplementary Fig. 4 The experimental concentration of JH-RE-06 was determined by IC50.** CCK-8 assays were used to measure the IC50 values (24 h) of JH-RE-06 in A549 and H1299 cells, and the dose-response curves are presented (n = 3). Based on these results, 3 μM and 5 μM were selected as the final experimental concentrations for A549 cells, while 5 μM and 7 μM were selected for H1299 cells.

**Supplementary Fig. 5 JH-RE-06 did not affect the expression of REV7 and REV3.** A549 and H1299 cells treated with DMSO or JH-RE-06 for 24h were harvested and analyzed by Western Blot (n = 3).

**Supplementary Fig. 6 JH-RE-06 has good safety *in vivo*. A** Body weight curves for the two groups. The data are shown as the mean body weights ± SEMs (n = 8 or 6 mice per group). **B** Representative images of HE staining of important organs (heart, liver, spleen, lung and kidney) from mice in the two groups. Scale bar: 50 μm. **C** Biochemical indexes in peripheral blood from the two groups of mice. No significant differences were found (n = 4).

**Supplementary Fig. 7 SERTAD2 is highly expressed in lung cancer and promotes the proliferation of lung cancer cells.** **A-C** The expression of SERTAD2 in TCGA, UALCAN and HPA database. *** *P* < 0.001. **D** SERTAD2 was successfully depleted via two different siRNAs (n = 3). **E** Knockdown of SERTAD2 significantly decreased the colony formation ability of lung cancer cells. *** *P* < 0.001 (n = 3). **F** The proliferation ability of A549 and H1299 cells was evaluated by an EdU incorporation assay. ** *P* < 0.01, *** *P* < 0.001 (n = 3). Scale bar: 50 μm.
