# Supplementary material for: REV1 promotes lung tumorigenesis by activating the Rad18/SERTAD2 axis
Source: Cell Death Dis. 2022 Feb 3;13(2):110. doi: 10.1038/s41419-022-04567-5 (PMC8814179; doi:10.1038/s41419-022-04567-5)
Supplement: Supplementary file 10 — Author Contribution Statement [file 41419_2022_4567_MOESM10_ESM.pdf]

DECLARATION OF CONTRIBUTIONS TO ARTICLE

ADMC

Manuscript Number: CDDIS-21-3834RR

Journal Name: *Cell Death & Disease* (the 'Journal')

Proposed Title of the Contribution:

REV1 promotes lung tumorigenesis by activating the Rad18/SERTAD2 axis (the 'Contribution)

Author(s):

Yunshang Chen, Xiaohua Jie, Biyuan Xing, Zilong Wu, Xijie Yang, Xinrui Rao, Yingzhuo Xu, Dong Zhou, Xiaorong Dong, Tao Zhang, Kunyu Yang, Zhenyu Li and Gang Wu (the 'Authors')

For all *CDDis* articles, each person named as an author in the published version must be able to show he or she has contributed substantially to the article.

Authorship credit should be based on 1) substantial contributions to conception and design, acquisition of data, or analysis and interpretation of data; 2) drafting the article or revising it critically for important intellectual content; and 3) final approval of the version to be published. Authors should meet conditions 1, 2 and 3.

Any person who cannot be shown to have made a substantial contribution to the article cannot be listed as an author in the final version. The name of any person who is deemed to have made a minor contribution can, however, appear in the Acknowledgments section of the article.

Please complete the table below to indicate the contributions of all named authors to the manuscript.

Author Full Name:	Specification of Contribution to the Manuscript:
Yunshang Chen	conception and design, acquisition of data, article drafting, final approval of the version to be published
Xiaohua Jie	conception and design, analysis of data, article drafting, final approval of the version to be published
Biyuan Xing	acquisition of data, article drafting, final approval of the version to be published
Zilong Wu	acquisition and analysis of data, final approval of the version to be published
Xijie Yang	acquisition and analysis of data, final approval of the version to be published
Xinrui Rao	analysis and interpretation of data, final approval of the version to be published
Yingzhuo Xu	analysis and interpretation of data, final approval of the version to be published
Dong Zhou	analysis of data, final approval of the version to be published
Xiaorong Dong	technical support, final approval of the version to be published
Tao Zhang	technical support, final approval of the version to be published
Kunyu Yang	technical support, final approval of the version to be published
Zhenyu Li	conception and design, article revising, final approval of the version to be published
Gang Wu	conception and design, acquisition of funding, article revising, final approval of the version to be published

Please complete the table below to indicate the contributions of all named authors to the figures.

Figure 1:

Gang Wu provided guidance and supervision.Yunshang Chen and Xiaohua Jie designed and performed the experiments. Biyuan Xing analyzed the data and prepared the figures.

Figure 2:

Zhenyu Li provided guidance and advice. Yunshang Chen and Zilong Wu performed the experiments. Xinrui Rao and Yingzhuo Xu analyzed the data and prepared the figures.

Figure 3:

Xiaorong Dong provided advice. Xiaohua Jie and Xijie Yang performed the experiments. Yunshang Chen analyzed the data. Dong Zhou prepared the figures.

Figure 4:

Kunyu Yang provided technical support. Yunshang Chen and Xiaohua Jie analyzed the sequencing data and performed experiments. Biyuan Xing drafted the figures.

Figure 5:

Tao Zhang provided advice. Xiaohua Jie designed the expriments.Yunshang Chen and XijieYang performed the experiments and prepared the figures.

Figure 6:

Gang Wu provided guidance and advice. Yunshang Chen and Biyuan Xing performed the experiments. Xiaohua Jie provided advice. Zilong Wu and Dong Zhou analyzed the data and prepared the figures.

Signed for and on behalf of the Author(s):	Print Name:	Date:
Gang Wu	Gang Wu	7th, Jan, 2022
